# The Ownership of Sanatoria

**Published:** 1899-12-16

**Authors:** 


### The Ownership of Sanatoria.

The somewhat portentous warning given by Dr. Heron, at a recent meeting of the Royal Medical and Chirurgical Society, in regard to the dire consequences which might happen should medicine and hotel management join hands in the person of the physician-proprietor-manager when sanatoria for the treatment of tuberculosis arise throughout the land, and the remarks made at a previous meeting about the evils of such institutions being run as hotels "with a physician attached," raise the question how such establishments will best obtain the medical supervision so necessary to their success.

There is no doubt that, if medical men are to be credited with all the vices, the running of a sanatorium for the treatment of consumption will offer extraordinary opportunities for the practice of the worst forms of quackery, and for screwing the uttermost farthing out of the unfortunate believers in the forms of treatment dealt in by these establishments. But we do not feel inclined to attribute such lax morality to the members of an honourable profession, and we put the suggestion aside all the more readily because we feel well assured that, if sanatorium treatment is allowed to take its proper place in England, the choice of the institution to which a patient is to be sent will always fall to outside medical men, than whom none could be more ready to put down a firm foot on the first indication of quackery.

The real objection felt to the physician-proprietor-manager among those who are deeply imbued with professional feeling is that it welds the link between medicine and commercialism, and tends still further to cut us adrift from the old position which looked upon the fee as an honorarium, and regarded all other money dealings between doctor and patient as absolutely wrong. It is the commercialism of the whole affair which stinks in the nostrils of the purists.

This is a side of the subject which is too large to enter into at the present moment, but it is perhaps worth pointing out that the question is not entirely one of conforming to accepted professional customs, but rather one of acting for the best under circumstances which are entirely new. It is not for consumption alone that sanatoria and "institutes" and "homes" are likely to arise. With the growth of specialism it has become clear, not only that a man who devotes himself to one department of medicine can attain a technical skill and a specialised knowledge beyond what is possible to general practitioners, but also that such a man can reach still higher levels if he does his work in hospitals or institutions devoted entirely to his own branch of practice. Such is probably the fact; such is certainly the opinion of the public. Germany and the United States are over-run with such institutions, owned and managed by medical men, and although it may freely be admitted that the system is one which is not altogether good, still it is sure to spread, and will have to be put up with and moulded for the best, as has already been done with specialism, of which indeed it is an almost inevitable development. The consumption sanatorium for paying patients is, indeed, but the forerunner of a host of other institutions in which patients will find themselves surrounded by all that is most up-to-date in the treatment of their special diseases; and if the profession, by throwing discredit on the personage we have spoken of as the physician-proprietor-manager, succeeds in handing these institutions over to the laity, to be run by lay bodies who will merely employ medical men as resident physicians, we shall have the system all the same, but instead of being its masters we shall be servants in its bondage.

There can hardly be a doubt that, so far as the outside profession is concerned, it will always seem a more "professional" proceeding to send a patient to be treated by Dr. So-and-So than to send him to undergo the treatment of the So-and-So Company, Limited—between doing which and sending a patient to be under some bone-setter or some "medical rubber" there seems but little to choose, unless the presence of a resident physician in the building may be taken as giving professional sanction to what would otherwise be an unprofessional arrangement. Nor must we consider the question merely as it affects the doctors. We have to ask in what way will the best results be secured to the patients, and in regard to this again we can but return one answer.

Notwithstanding all that can be urged against the intrusion of commercialism and of large questions of finance into the life of the medical practitioner, we can but feel that more good work will be done in an institution owned and managed by a responsible physician, who measures his success by the professional position which he is able to attain, than in one in which the physician is merely a paid officer, liable to dismissal if the régime he enforces should not prove attractive to the guests. The physician-proprietor-manager may not be ideal, but he is probably the best solution of a problem surrounded by many difficulties.

